# Supplementary material for: Harnessing interpretable deep learning to predict resistance in Klebsiella pneumoniae
Source: Front Cell Infect Microbiol. 2026 May 28;16:1859508. doi: 10.3389/fcimb.2026.1859508 (PMC13253382; doi:10.3389/fcimb.2026.1859508)
Supplement: Supplementary file 1 [file DataSheet1.pdf]

Supplementary information for **Harnessing Interpretable Deep Learning to Predict Resistance in *Klebsiella pneumoniae***

Nicolas da Matta Freire Araujo[1], Márcia da Silva Chagas[1], Mateus Fernandes Santos[2], Renata Freire Alves Pereira[3,4], Rafaela Correia Brum[5], Felipe Ramos Pinheiro[3,6], Melise Chaves Silveira[7], Felicita Mabel Duré[1,8], Beatriz de Lima Alessio Müller[9], Audrien Alves Andrade de Souza[9], Alessandra Beatriz Santos Rondon Souza[4,10], Ágatha Ferreira de Souza[10], Ana Paula D'Alincourt Carvalho-Assef[11], Aline dos Santos Moreira[9], Marcelo Trindade dos Santos[12], Adriano Maurício de Almeida Côrtes[2,13], Bruno de Araújo Penna[10], Thiago Pavoni Gomes Chagas[3,14], Fábio Aguiar-Alves[3,4,6,15], Fabrício Alves Barbosa da Silva[1,*]

1 - Scientific Computing Program, Oswaldo Cruz Foundation (FIOCRUZ), Rio de Janeiro, Brazil
2 - Department of Applied Mathematics, Institute of Mathematics, Federal University of Rio de Janeiro (UFRJ), Rio de Janeiro, 21941-909, Brazil
3 - Laboratory of Molecular Epidemiology and Biotechnology, School of Pharmacy/Fluminense Federal University, Mario Viana, 523 - Niteroi/RJ, 2424.241-000, Brazil.
4 - Graduate Program in Microbiology and Parasitology. Biomedical Institute - Fluminense Federal University, Valonguinho Campus, Centro, Niteroi/RJ, 24020-140, Brazil.
5 -Department of System Engineering and Computation, State University of Rio de Janeiro, Rio de Janeiro, Brazil
6 - Graduate Program in Pathology, Fluminense Federal University, Niteroi /RJ, Brazil.
7 - Bioinformatics Laboratory, National Laboratory for Scientific Computing (LNCC), Petrópolis, Brazil
8 - Central Public Health Laboratory (LCSP), Ministry of Public Health and Social Welfare MSPyBS, Asunción, Paraguay
9 - Laboratory of Applied Genomics and Bioinnovations - IOC/FIOCRUZ, Next-Generation Sequencing Platforms IOC/RPT01J - Network of Technological Platforms/FIOCRUZ, Brazil
10 - Laboratory of Gram-Positive Cocci / Fluminense Federal University, Niteroi/RJ, Brazil.
11 - Laboratory of Bacteriology Applied to One Health and Antimicrobial Resistance, Oswaldo Cruz Foundation (FIOCRUZ), Rio de Janeiro, Brazil
12 - Department of Computational Modeling, National Laboratory for Scientific Computing (LNCC), Petrópolis, Brazil
13 - Systems Engineering and Computer Science Program, Coordination of Postgraduate Programs in Engineering (COPPE), Federal University of Rio de Janeiro (UFRJ), Rio de Janeiro, 21941-972, Brazil
14 - Department of Pathology, School of Medicine, Fluminense Federal University, Niteroi/RJ, Brazil.
15 - Department of Pharmaceutical Sciences, Lloyd L. Gregory School of Pharmacy, Palm Beach Atlantic University – USA.

*To whom correspondence should be addressed (fabricio.silva@fiocruz.br)

Table S1 - Validation and test set results for meropenem

	Fold	ROC AUC	BACC	F1 score	MCC
Validation	1	0,93	0,91	0,9	0,81
Validation	2	0,95	0,9	0,9	0,8
Validation	3	0,89	0,83	0,83	0,67
Validation	4	0,87	0,83	0,83	0,67
Validation	5	0,94	0,87	0,88	0,75
Test	1	0,82	0,81	0,78	0,63
Test	2	0,93	0,86	0,86	0,72
Test	3	0,75	0,74	0,72	0,49
Test	4	0,74	0,75	0,72	0,51
Test	5	0,8	0,74	0,75	0,47

Table S2 - Statistics for meropenem

		ROC AUC	BACC	F1 score	MCC
Average (Validation)	All Folds	0,916	0,868	0,868	0,74
Average (Test)	All Folds	0,808	0,78	0,766	0,564
Std Dev (Validation)	All Folds	0,034	0,038	0,036	0,068
Std Dev (Test)	All Folds	0,076	0,053	0,058	0,107

Table S3 - Validation and test set results for cefepime

	Fold	ROC AUC	BACC	F1 score	MCC
Validation	1	0,79	0,71	0,74	0,42
Validation	2	0,71	0,66	0,71	0,32
Validation	3	0,78	0,72	0,76	0,43
Validation	4	0,81	0,73	0,76	0,45
Validation	5	0,79	0,73	0,76	0,46
Test	1	0,83	0,76	0,79	0,51
Test	2	0,8	0,74	0,76	0,47
Test	3	0,81	0,72	0,75	0,43
Test	4	0,78	0,69	0,72	0,37
Test	5	0,8	0,71	0,77	0,42

Table S4 - Statistics for cefepime

		ROC AUC	BACC	F1 score	MCC
Average (Validation)	All Folds	0,776	0,71	0,746	0,416
Average (Test)	All Folds	0,804	0,724	0,758	0,44
Std Dev (Validation)	All Folds	0,038	0,029	0,022	0,056
Std Dev (Test)	All Folds	0,018	0,027	0,026	0,053

Table S5 - Validation and test set results for ceftazidime

	Fold	ROC AUC	BACC	F1 score	MCC
Validation	1	0,94	0,87	0,92	0,66
Validation	2	0,93	0,88	0,93	0,67
Validation	3	0,92	0,87	0,93	0,67
Validation	4	0,92	0,87	0,9	0,63
Validation	5	0,96	0,88	0,92	0,67
Test	1	0,88	0,88	0,94	0,72
Test	2	0,9	0,83	0,92	0,61
Test	3	0,91	0,85	0,93	0,67
Test	4	0,92	0,85	0,9	0,6
Test	5	0,92	0,87	0,93	0,68

Table S6 - Statistics for ceftazidime

		ROC AUC	BACC	F1 score	MCC
Average (Validation)	All Folds	0,934	0,874	0,92	0,66
Average (Test)	All Folds	0,906	0,856	0,924	0,656
Std Dev (Validation)	All Folds	0,017	0,005	0,012	0,017
Std Dev (Test)	All Folds	0,017	0,019	0,015	0,050

Table S7 - Validation and test set results for gentamicin

	Fold	ROC AUC	BACC	F1 score	MCC
Validation	1	0,94	0,89	0,89	0,78
Validation	2	0,9	0,85	0,84	0,71
Validation	3	0,89	0,83	0,82	0,66
Validation	4	0,96	0,89	0,89	0,79
Validation	5	0,9	0,82	0,82	0,64
Test	1	0,89	0,81	0,81	0,62
Test	2	0,91	0,75	0,77	0,51
Test	3	0,74	0,71	0,73	0,42
Test	4	0,86	0,75	0,75	0,51
Test	5	0,89	0,8	0,8	0,59

Table S8 - Statistics for gentamicin

		ROC AUC	BACC	F1 score	MCC
Average (Validation)	All Folds	0,918	0,856	0,852	0,716
Average (Test)	All Folds	0,858	0,764	0,772	0,53
Std Dev (Validation)	All Folds	0,030	0,033	0,036	0,068
Std Dev (Test)	All Folds	0,068	0,041	0,033	0,078
